# Efficacy of cutting balloon angioplasty versus high-pressure balloon angioplasty for the treatment of arteriovenous fistula stenoses in patients undergoing hemodialysis: Systematic review and meta-analysis

**DOI:** 10.1371/journal.pone.0296191

**Published:** 2024-01-25

**Authors:** Shuyue Pang, Tianying Chang, Mingxin Chang, Xu Huang, Xiaodan Wang, Meijin Song, Zhongtian Wang, Shoulin Zhang

**Affiliations:** 1 College of Traditional Chinese Medicine, Changchun University of Chinese Medicine, Changchun Jilin, China; 2 EBM Office, The Affiliated Hospital to Changchun University of Chinese Medicine, Changchun Jilin, China; 3 Nephrology Department, The Affiliated Hospital to Changchun University of Chinese Medicine, Changchun Jilin, China; BSMMU: Bangabandhu Sheikh Mujib Medical University, BANGLADESH

## Abstract

This systematic review and meta-analysis aimed to assess and compare the therapeutic outcomes of cutting balloon angioplasty and high-pressure balloon angioplasty for arteriovenous fistula stenosis in hemodialysis patients. All studies indexed in PubMed, Embase, and Cochrane Library Web of Science were retrieved. The retrieval deadline was July 15, 2023. Risk of bias 2.0 was used to evaluate the quality of the included studies. Revman 5.4 software was used for data analysis. This review included three studies and 180 patients, with 90 patients in the cutting balloon angioplasty group and 90 patients in the high-pressure balloon angioplasty group. The results of the meta-analysis suggested that compared with high-pressure balloon angioplasty, cutting balloon angioplasty can improve primary lesion patency rates of internal arteriovenous fistulas at 6 months (relative risk, 1.45; 95% confidence interval, 1.08–1.96; P = 0.01). However, there were no significant differences between the technical success rate (relative risk, 0.99; 95% confidence interval, 0.93–1.05; P = 0.72) and clinical success rate (relative risk, 1.01; 95% confidence interval, 0.95–1.07; P = 0.73). Therefore, cutting balloon angioplasty is likely to increase primary lesion patency rates at 6 months. However, more high-quality, large-sample, multicenter, randomized controlled trials are needed for further validation due to the limited number of included studies.

## Introduction

Recently, there has been an annual increase in the number of patients requiring long-term hemodialysis (HD) due to chronic renal failure [1]. However, vascular access is an essential condition for HD and serves as the “lifeline” of patients [2]. Arteriovenous fistulas for vascular access have the advantages of low infection rates, low incidence of thrombotic complications, and longer use times; therefore, autologous arteriovenous fistula are established in more than 95% of the population requiring HD [3, 4]. However, many factors such as underlying diseases, hemodynamics, and puncture injury can affect internal arteriovenous fistulas. Patients often develop stenosis of the internal fistula due to factors such as intimal hyperplasia, thrombogenesis, and occlusion, resulting in loss of the fistula function [5, 6]. Previously, patients with narrow arteriovenous fistulas were treated with surgical resection and lesion reconstruction. However, with the development of interventional radiology, minimally invasive interventions that have resulted in satisfactory curative effects have been introduced. Nonetheless, interventional radiology involves some challenges, including the need for large equipment and damage caused by contrast agents and radiation. These factors, to some extent, limit its clinical application. Therefore, based on experience with minimally invasive interventions, it was determined that color ultrasound, which is useful and convenient, can achieve good curative effects. Recently, color ultrasound-guided percutaneous balloon dilation angioplasty has become a new treatment method for arteriovenous fistula stenosis. Under ultrasound guidance, percutaneous balloon dilation angioplasty can monitor the function and structure of internal fistula vessels in real-time and in multiple dimensions [7]. Considering its shallow location, the vascular structure and instrumental characteristics can be clearly displayed. In particular, Doppler ultrasound has great advantages when used to evaluate hemodynamics, thereby improving the accuracy of dilation. Percutaneous balloon dilatation angioplasty is the first-line treatment for patients with arteriovenous fistula stenosis [8]. Cutting-balloon dilatation is characterized by low-pressure directional cutting dilatation, and the surface of the balloon comprises three to four cutting blades. During expansion, the blades can cut the lesion site in a directional manner and decrease the pressure needed for balloon dilatation and the damage caused by thrombosis, thereby delaying restenosis caused by intimal hyperplasia [9, 10]. Residual stenosis is a crucial factor resulting in poor outcomes of percutaneous transluminal angioplasty. When dealing with intractable stenosis, regular or high-pressure balloons may fail to achieve full expansion, resulting in incomplete dilation. Residual stenosis more than 30% is considered a technical failure that obviously affects the patency rate. Using a cutting balloon can address residual stenosis resulting from incomplete balloon expansion [11]. A high-pressure balloon, delivering 24 to 36 Pa of dilatation pressure, can mechanically disrupt the dense fibrous tissue in the stenotic segment, thus enhancing the success rate of percutaneous balloon dilatation angioplasty in treating these lesions. Studies have suggested that the patency rate of high-pressure balloon treatment for refractory arteriovenous fistula stenosis is approximately 39% to 43% six months after surgery [12, 13]. High-pressure balloons have been widely applied for the treatment of refractory arteriovenous fistula stenosis [14]; however, whether to use a high-pressure balloon or a cutting balloon for patients with stenosis of the internal arteriovenous fistula remains controversial [15]. Therefore, this study aims to address this controversy by conducting a meta-analysis and systematic review and providing new options for patient treatment.

## Materials and methods

The systematic review was accepted by the online PROSPERO international prospective register of systematic reviews [16] of the National Institute for Health Research (CRD42023423658).

### Literature retrieval

A systematic search of studies comparing cutting balloon angioplasty and high-pressure balloon angioplasty was conducted by two authors using the PubMed, Embase, Cochrane Library, and Web of Science databases. Angioplasty studies on the efficacy of treating arteriovenous fistula stenosis in hemodialysis patients were retrieved using a combination of subject and free words for retrieval. The search terms "cutting balloon angioplasty," "high-pressure balloon angioplasty," "hemodialysis," and "arteriovenous fistula" were employed.

### Inclusion and exclusion criteria

Adults undergoing HD with arteriovenous fistula stenosis who were eligible for cutting balloon angioplasty performed as the intervention measure (the experimental group) or high-pressure balloon angioplasty as the intervention method (the control group), were considered for inclusion in this analysis. The secondary outcomes included technical and clinical success rates. Technical success was was defined as achieving less than 30% residual diameter stenosis as evident in the final angiogram after angioplasty. Clinical success was defined as at least one successful session of hemodialysis using the arteriovenous fistulas after angioplasty. Duplicate studies, animal studies, protocols, systematic reviews, conference abstracts, articles without available full-text, and articles lacking data were excluded from our analysis.

### Data extraction

Two authors carefully screened the literature according to the inclusion and exclusion criteria. If conflicts occurred, then both parties reached an agreement through consultation or sought advice from a third party. The information extracted from the included studies were author names, publication year, region, intervention, number of samples (trial and control groups), age, sex, mean fistula age, number of radiocephalic fistulas, and number of brachiocephalic fistulas.

### Quality evaluation

Two researchers independently used the Cochrane Collaboration tool to assess the risk of bias as low, unclear, or high [17]. The reviewed content included the following: random sequence generation (selective bias), assignment hiding (selective bias), implementor and participant blinding (implementation bias), outcome evaluator blinding (observation bias), integrity of final data (follow-up bias), selective reporting of findings (reporting bias), and other potential sources of bias. Each study was individually evaluated according to the criteria. If a study fully met all criteria, it was categorized as low-risk, indicating high quality and low overall bias risk. Conversely, if a study did not meet the criteria, it was classified as high-risk, signifying low quality and high overall bias risk. The evaluation of outcome uses gradeprofile which listed in Table 1. The PRISMA 2020 Main Checklist can be found in the S1 Checklist.

**Table 1:** Grade profiler.

Outcome	Grade
Primary lesion patency rates at 6 months	Low
Technical success rate	Low
Clinical success rate	Low

### Data analysis

Revman 5.4 was used to perform statistical analyses. Heterogeneity among the included studies was assessed using I^2^ values or Q statistics. I^2^ values of 0%, 25%, 50%, and 75% indicated no heterogeneity, low heterogeneity, medium heterogeneity, and high heterogeneity, respectively. When I^2^ ≥50%, a sensitivity analysis was performed to explore the source of heterogeneity. If heterogeneity was less than 50%, then the fixed effect model was used for analysis. A random-effects model and Egger test were used to evaluate publication bias.

## Results

### Literature retrieval results

**Fig 1:**
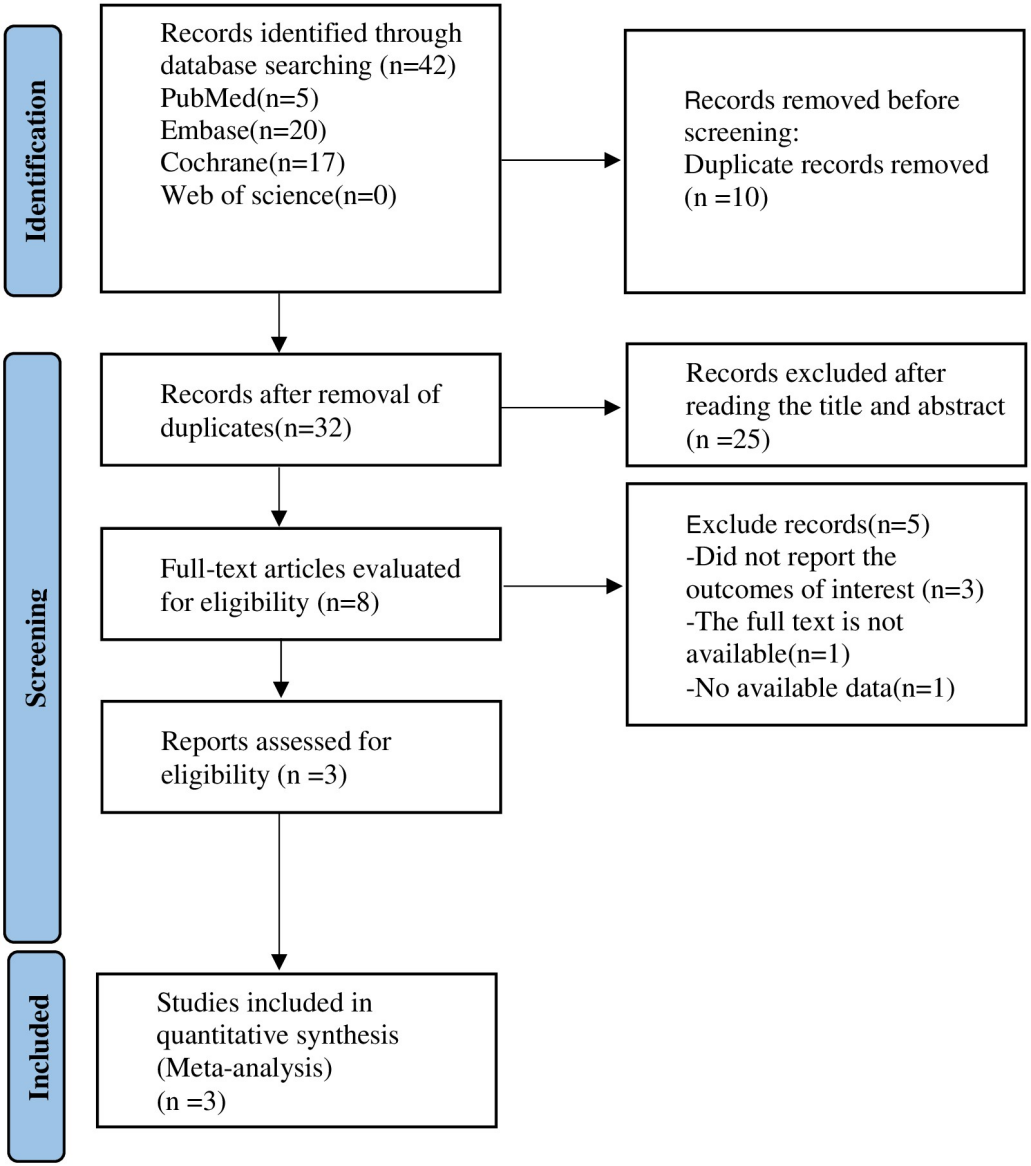
PRISMA flow diagram of the study process. PRISMA, Preferred Reporting Items for Systematic review and Meta-analysis.

During the preliminary retrieval process, 42 studies were obtained, and 10 duplicate studies were removed. Twenty-five literatures were excluded after reading their titles and abstracts. Five studies were removed after reading the full text. Finally, three [18–20] randomized controlled trials were included. The literature retrieval flow is displayed in Fig 1.

### Quality evaluation of basic features of the included studies

**Fig 2:**
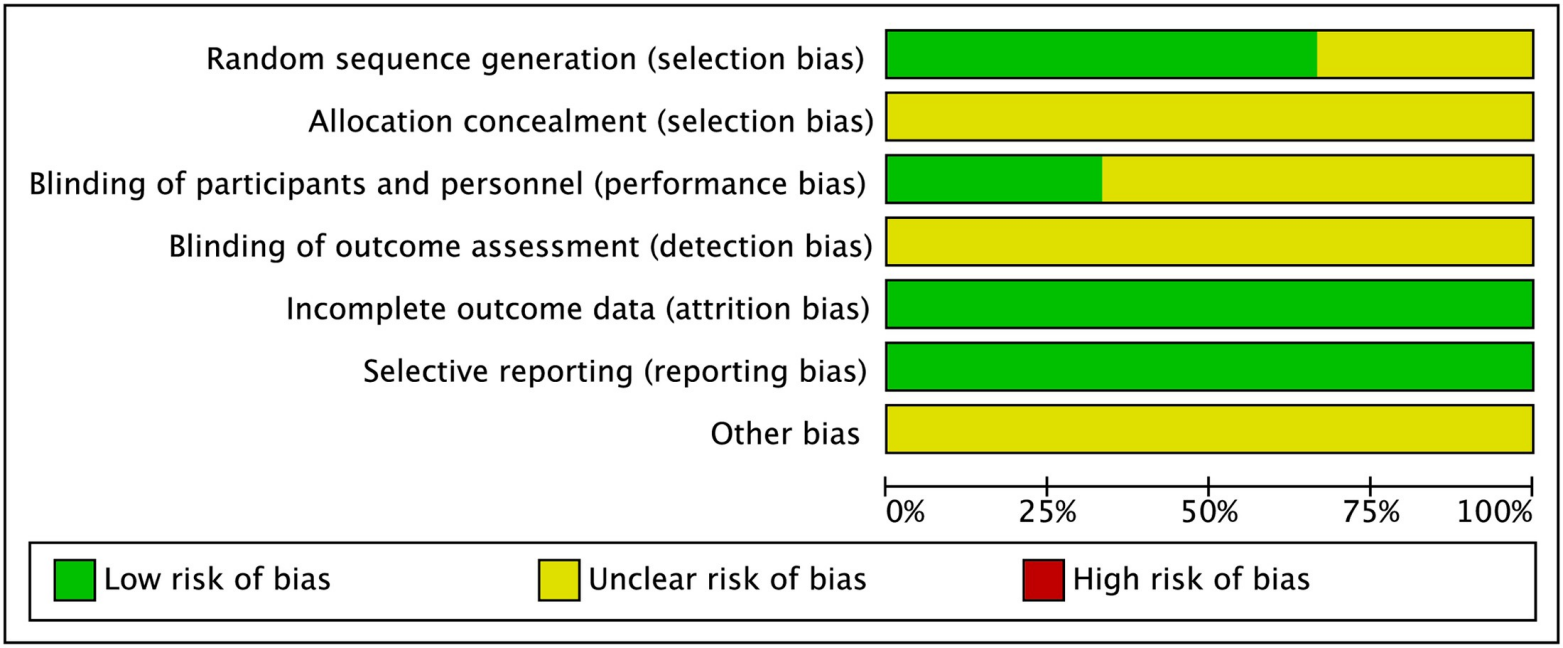
Risk of bias.

**Fig 3:**
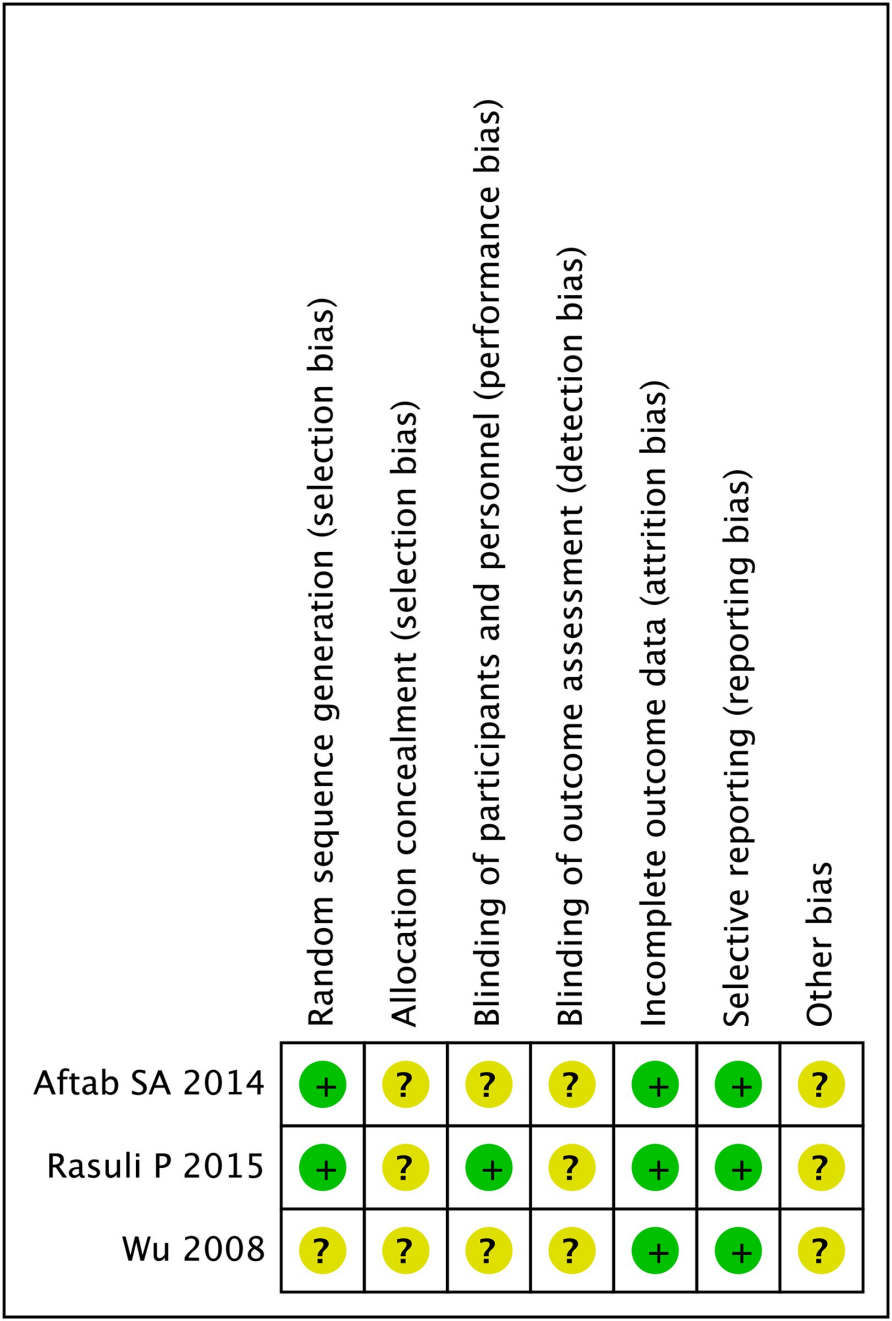
Summary of the risk of bias.

Three studies, comprising 180 patients aged 61 to 70 years, were included. Of these, 90 patients had undergone cutting balloon angioplasty and 90 patients had undergone high-pressure balloon angioplasty. The characteristics of these studies are listed in Table 2. One of the included studies did not mention the specific randomization methods in detail; therefore, the evaluation was not clear. Most of the included methods were not clearly explained. The specific quality evaluation is shown in Figs 2 and 3.

**Table 2:** Characteristics of the included studies.

Author	Region	Intervention		patients		Age		Sex(male)		Mean fistula age		Radiocephalic, n (%)		brachiocephalic n (%)	
		EG	CG	EG	CG	EG	CG	EG	CG	EG	CG	EG	CG	EG	CG
Rasuli 2015	Canada	cutting balloon angioplasty	high-pressure balloon angioplasty	19	20	61	70	11	11	NA	NA	10	7	9	13
Wu 2008	Taiwan	cutting balloon angioplasty	high-pressure balloon angioplasty	35	35	62.5 ±15.1	61.0 ±11.7	18	25	26.3±26.9	36.0±42.1	24 (68)	25 (71)	11 (31)	10 (28)
Aftab 2014	Singapore	cutting balloon angioplasty	high-pressure balloon angioplasty	36	35	62.50±10.84	57.60±11.58	24	25	23.41±19.11	21.00±15.50	17 (47.2)	9 (25.7)	15 (41.7)	19 (54.3)

### Meta-analysis results

#### Primary lesion patency rates at 6 months

**Fig 4:**
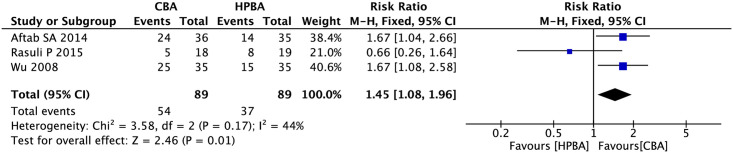
Forest plot of primary lesion patency rates at 6 months.

Three studies reported primary lesion patency rates at 6 months and included 89 patients in the cutting balloon angioplasty group and high-pressure balloon angioplasty group (heterogeneity: I^2^ = 44%, P = 0.17). Therefore, a fixed-effects model was used for the analysis. The analysis results indicated that compared with high-pressure balloon angioplasty, cutting balloon angioplasty could improve primary lesion patency rates at 6 months (relative risk [RR], 1.45; 95% confidence interval [CI], 1.08–1.96; P = 0.01; Fig 4).

#### Technical success rate

**Fig 5:**
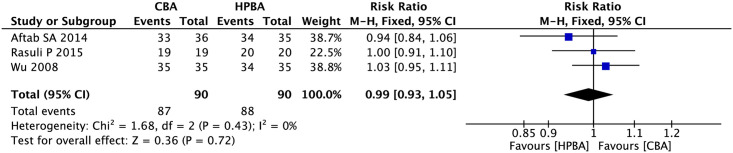
Forest plot of technical success rates.

Three studies mentioned the technical success rate and included 90 participants in the cutting balloon angioplasty group and the high-pressure balloon angioplasty group (heterogeneity: I^2^ = 0%, P = 0.43). Therefore, we adopted a fixed-effects model for our analysis. The analysis results indicated that there were no significant differences in the technical success rates of high-pressure balloon angioplasty and cutting balloon angioplasty for arteriovenous fistulas (RR, 0.99; 95% CI, 0.93–1.05; P = 0.72; Fig 5).

#### Clinical success rate

**Fig 6:**
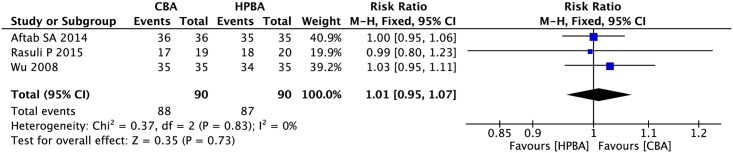
Forest plot of clinical success rates.

Three studies mentioned the clinical success rate and included 90 participants in the cutting balloon angioplasty group and the high-pressure balloon angioplasty group (heterogeneity: I^2^ = 0%, P = 0.83). Therefore, we adopted a fixed-effects model for our analysis. The analysis results indicated that there were no significant differences in the technical success rates of high-pressure balloon angioplasty and cutting balloon angioplasty for arteriovenous fistulas (RR, 1.01; 95% CI, 0.95–1.07; P = 0.73; Fig 6).

#### Publication bias

**Fig 7:**
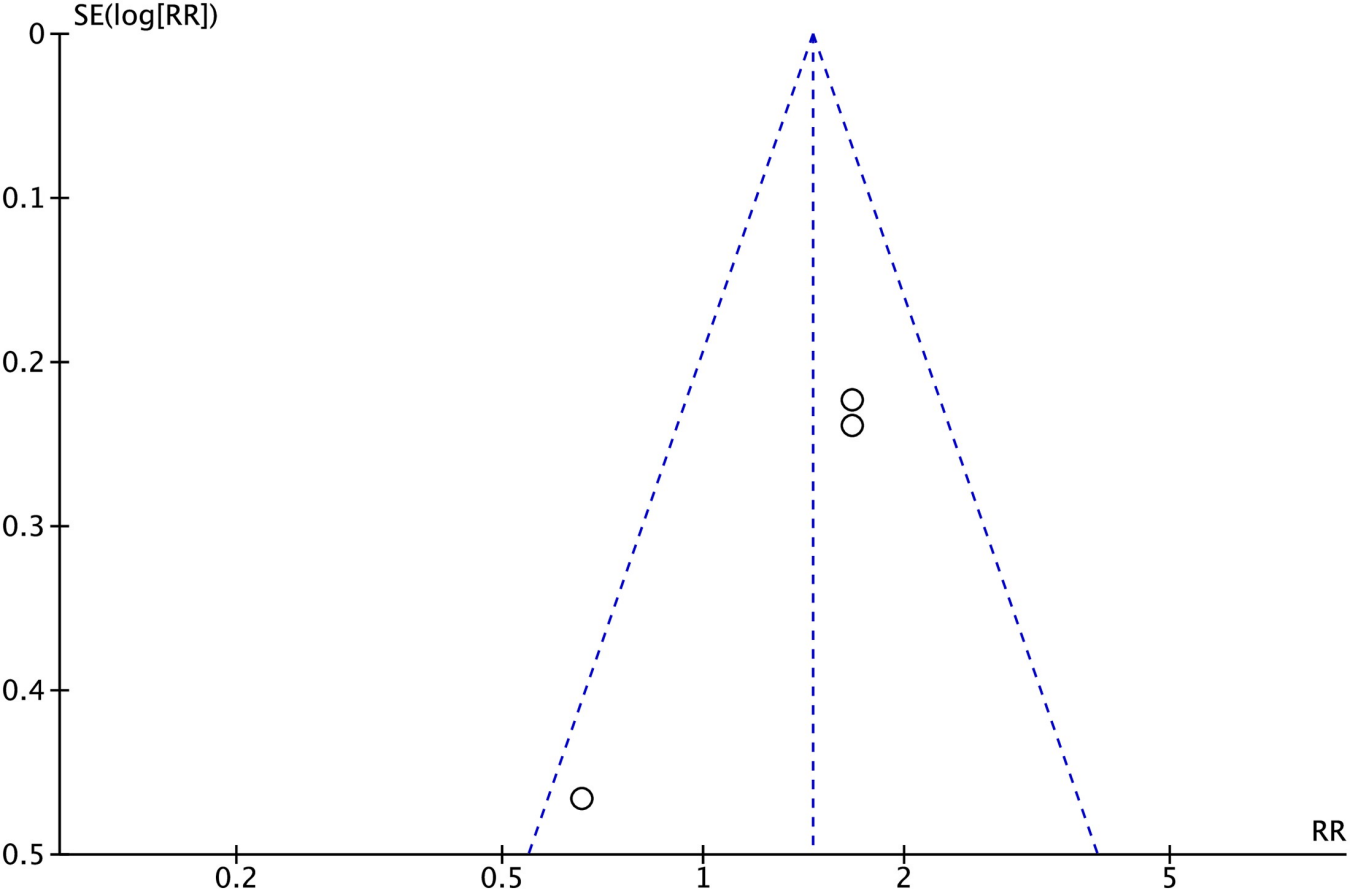
Funnel plot of primary lesion patency rates at 6 months.

**Fig 8:**
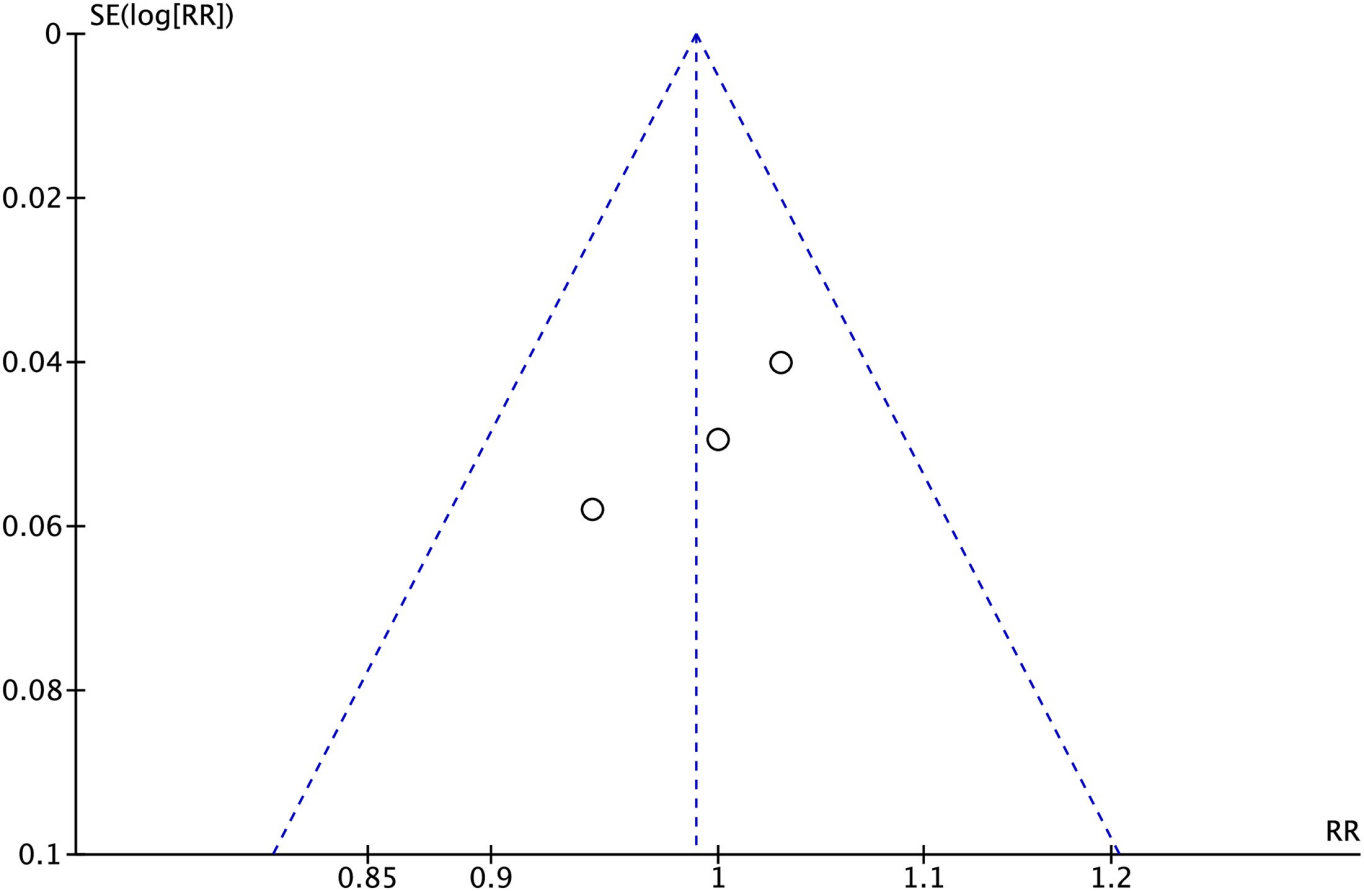
Funnel plot of technical success rates.

**Fig 9:**
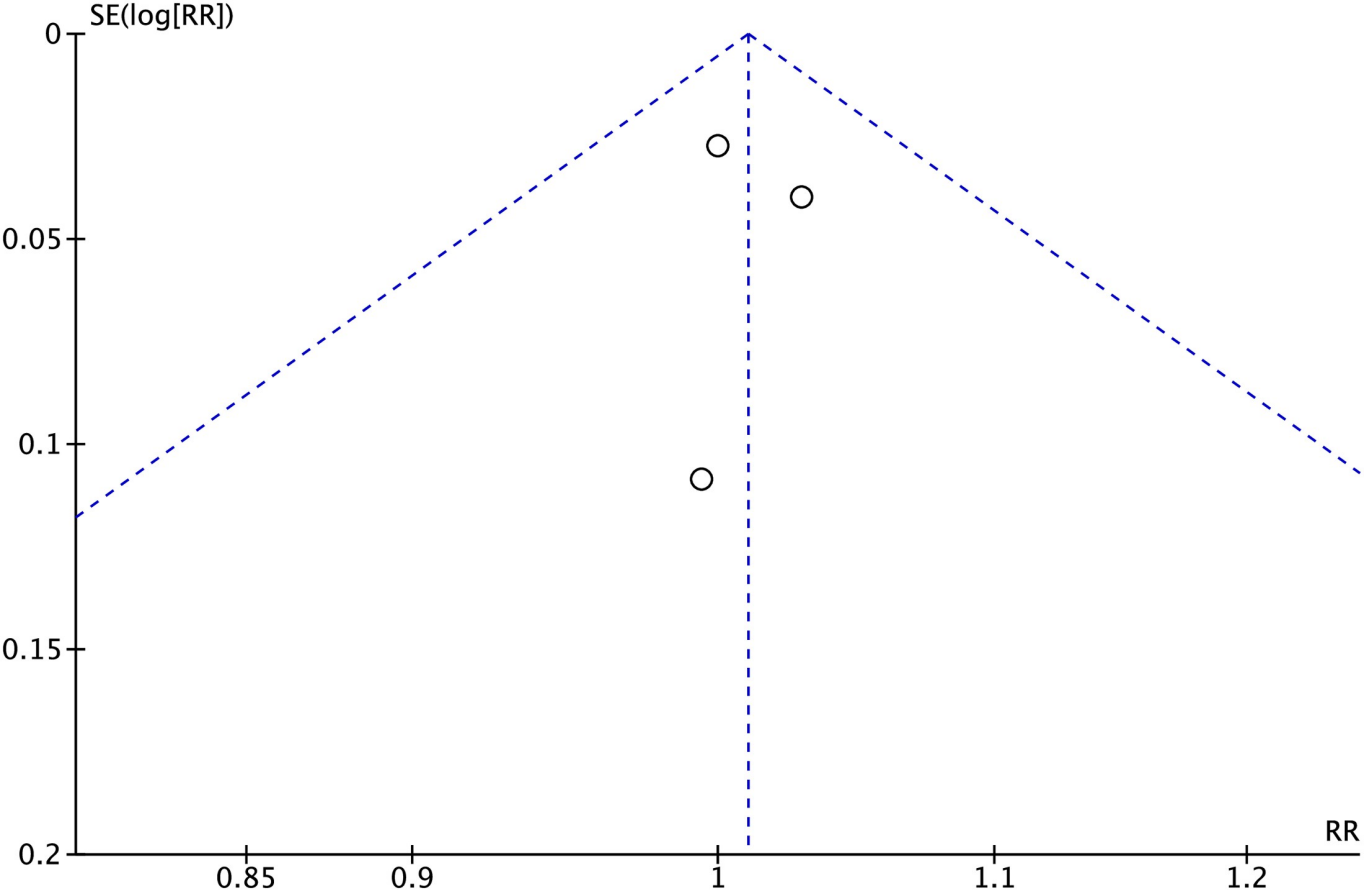
Funnel plot of clinical success rates.

To evaluate publication bias for primary lesion patency rates at 6 months, as well as the technical and clinical success rates, funnel plots were utilized. The results indicated a high probability of deviation in primary lesion patency rates at 6 months, as well as in the technical and clinical success rates (Figs 7–9).

## Discussion

This is the first meta-analysis to compare the efficacy of cutting balloon angioplasty and high-pressure angioplasty for arteriovenous fistula stenosis in patients undergoing HD. This study found that cutting balloon angioplasty was likely to improve primary lesion patency rates at 6 months. This finding is in agreement with the conclusion of a previous study [21] that found that cutting balloon angioplasty was more effective than high-pressure balloon angioplasty for patients undergoing HD with arteriovenous fistula stenosis, as indicated by the primary lesion patency rates at 6 months. In contrast, Rasuli [20] found that the patency rate of stenotic vessels at 6 months after high-pressure balloon angioplasty was higher than that after cutting balloon angioplasty. Various conclusions regarding the effectiveness of HD pathway stenosis treatment have been reported, with differences possibly attributed to factors such as the method of treating blood vessel stenosis and the type of HD pathway used during follow-up. Therefore, large-scale clinical trials with extended follow-up times are needed for further exploration [22, 23]. Barath [24] proposed that cutting balloon angioplasty has advantages over high-pressure angioplasty for maintaining vascular patency and assumed that a controlled directional incision could be performed on the vascular wall at the stenosis to reduce the force required to effectively expand the stenosis. They reported that this approach reduced the vascular wall damage, decreased the reaction to neointimal hyperplasia, and extended the patency time. These study results align with the theory, suggesting that cutting balloon angioplasty leads to longer patency compared to high-pressure angioplasty [25, 26].

The loss of the HD pathway function was mainly caused by anastomotic stenosis and thrombosis. There are many causes of vascular stenosis, including intimal hyperplasia, vascular remodeling, smooth muscle cell migration, and vascular contraction [27]. Repeated vascular puncture during HD may enhance inflammation and promote stenosis [28]. Vascular stenosis usually refers to a lumen stenosis of more than 50%. When HD fails to reach the expected blood flow or when hemodynamic parameters show stenosis of the internal fistula vessel, positive intervention treatments are required [29]. Vorwerk [30] used cutting balloon angioplasty to treat stenosis to allow HD access as early as possible. Three or four blades were mounted lengthwise on the cutting balloon and passed lengthwise through the surface of the cutting balloon. Under local anesthesia, through a small surgical incision and digital subtraction blood tube imaging or color Doppler ultrasound guidance, cutting balloons filled with air created an incision and released the circumferential pressure. At low pressure, cutting and inflating the balloon promoted more uniform angiectasis, reducing the risk of widespread air pressure injury and maximizing vascular resource protection. Cutting balloon angioplasty under low pressure can reduce the inflammatory response and the risks of cell proliferation and restenosis [31]. However, this study found that cutting balloon angioplasty and high-pressure angioplasty were not significantly different in terms of the technical and clinical success rates, possibly due to the heterogeneity among the studies and limited number of patients included. Furthermore, heterogeneity was likely induced by variations in equipment and surgical methods employed throughout the studies.

### Limitations

This study had some limitations. This meta-analysis included a small number of studies and cases, and the estimate of publication bias was insufficient. Some studies did not provide detailed data regarding follow-up, and the number of events was estimated from survival curves or ratios in the literature, which may not accurately represent the actual number. Additionally, other underlying diseases, dialysis duration, lesion type and location, and other characteristics of the cases were not further classified; therefore, further analyses may help clarify the heterogeneity of these results. However, it is noteworthy that although cutting balloon angioplasty can increase primary lesion patency rates at 6 months, there were no significant differences in the technical and clinical success rates of high-pressure balloon angioplasty and cutting balloon angioplasty. Therefore, caution is required when interpreting these results.

## Conclusions

Based on recent findings, cutting balloon angioplasty could increase primary lesion patency rates at 6 months. However, because of the limited number of included studies, more high-quality, large-sample, multicenter, randomized controlled trials are required to validate these findings.

## Supporting information

S1 Checklist PRISMA 2020 main checklist [32]. (PDF)

S1 Data (XLSX)
